# Low serum HDL-cholesterol is associated with increased risk of the subcortical small vessel type of dementia

**DOI:** 10.1016/j.cccb.2024.100229

**Published:** 2024-06-05

**Authors:** Elin Axelsson Andrén, Dewa Safi, Anders Wallin, Johan Svensson

**Affiliations:** a Department of Internal Medicine and Clinical Nutrition, Institute of Medicine, Sahlgrenska Academy, University of Gothenburg, Gothenburg, Sweden; b Department of Psychiatry and Neurochemistry, Institute of Neuroscience and Physiology, Sahlgrenska Academy, University of Gothenburg, Mölndal, Sweden; c Region Västra Götaland, Department of Internal Medicine, Skaraborg Central Hospital, Skövde, Sweden

**Keywords:** HDL-cholesterol, Triglycerides, Lipid pattern, Subcortical small vessel type of dementia, Alzheimer's disease, Mixed dementia

## Abstract

**Background:**

There are conflicting results whether serum lipid pattern is related to the amount of white matter hyperintensities (WMHs) on magnetic resonance imaging. Little is known of the associations between lipid concentrations and the subsequent risk of the subcortical small vessel type of dementia (SSVD), in which WMHs are a prominent manifestation. Here, we determined whether lipid levels are associated with the risk of SSVD, Alzheimer's disease (AD), or mixed dementia (combined AD and SSVD).

**Methods:**

This was a longitudinal, prospective study of 329 patients with subjective or objective mild cognitive impairment at baseline. The statistical analyses included Cox proportional hazards regression with adjustments for age, gender, education, body mass index, current smoking, hypertension, diabetes mellitus, and *APOE* ε4 genotype.

**Results:**

During the follow-up (mean 4.1 years), 80 patients converted to dementia [SSVD, *n* = 15 (5 %); AD, *n* = 39 (12 %); and mixed dementia, *n* = 26 (8 %)]. Serum high-density lipoprotein cholesterol (HDL, per SD increase) was inversely associated with the risk of SSVD, whereas triglycerides (TG), low-density lipoprotein cholesterol (LDL)/HDL ratio, and TG/HDL ratio were positively associated with SSVD risk. Furthermore, the lowest HDL tertile was associated with a sevenfold, and the highest tertile of TG/HDL ratio with a threefold, increase in SSVD risk. There were no associations with the risk of AD or mixed dementia after adjustment for covariates.

**Conclusion:**

In a memory clinic population, low HDL and high TG/HDL ratio were independent risk factors of SSVD, but not of AD or mixed dementia.

## Highlights

• A longitudinal mono-center study of 329 patients at a memory clinic.
• At baseline, patients had subjective or objective mild cognitive impairment.
• Serum HDL was inversely associated with the risk of SSVD.
• The lowest HDL tertile was associated with a sevenfold increase in SSVD risk.
• Lipid levels were unrelated to the risk of Alzheimer's disease or mixed dementia.

## 1 Introduction

Vascular cognitive impairment is a common but heterogenous age-related cognitive disorder [[1], [2], [3]]. After large vessel strokes, there is a varying progression from the primary event until the manifestation of vascular dementia (VaD) [[1], [2], [3], [4]]. In contrast, in the subcortical small vessel type of dementia (SSVD), there is a gradual progress over many years before the manifestation of dementia [5,6]. Characteristics of SSVD include impaired speed and attention, executive dysfunction, deteriorations of motor functions and mood, and mild to moderate memory impairment, and these disease features correlate with subcortical vascular pathologies like white matter hyperintensities (WMHs) on magnetic resonance imaging (MRI) [1,7]. Moreover, in mixed dementia, cerebral vascular changes coexist with Alzheimer's disease (AD) neuropathology [1,2,8].

Risk factors for VaD development include higher age as well as deteriorated cardiometabolic status in midlife [4,9,10], and after stroke, 20–25 % of the patients develop delayed dementia [4,11]. However, late in life, the associations between disturbed vascular risk factors and VaD risk have been less marked [10,[12], [13], [14]]. In a review and meta-analysis, there were no associations between late-life measurements of cholesterol, high-density lipoprotein cholesterol (HDL), or triglycerides (TG) and the risk of VaD [10] as classified mostly using NINDS-AIREN [15]. Also in terms of the risk of AD, cardiovascular risk factors evaluated in midlife have been more markedly associated with AD risk [9,13,[16], [17], [18]] compared with late life evaluations [13,14,16,17]. This could be in some accordance with the findings that body weight and serum cholesterol levels decline in prodromal AD [12,16,17,19]. Finally, in clinically established dementia, metabolic comorbidities have been more prominent in VaD patients than in AD patients [[20], [21], [22]].

In population-based studies, in which the amount of WMHs have been used as a proxy of SSVD, hypertension has been associated with WMH volume [[23], [24], [25]]. Impaired glucose homeostasis, diabetes mellitus (henceforth: diabetes), and obesity have been related to the amount of WMHs in some of the studies [[23], [24], [25], [26], [27]]. In terms of lipids, the results of population-based studies have been inconsistent [26]. Some studies have even found inverse relationships between levels of total cholesterol (TC) or low-density lipoprotein cholesterol (LDL) and the amount of WMH lesions on MRI [[28], [29], [30]]. In the only clinical study of a VaD population consisting solely of SSVD patients, serum levels of TC and LDL were lower in the SSVD patients than in the healthy controls [22].

In summary, it has previously not been evaluated in a memory clinic population whether serum lipid pattern is related to the risk of clinically diagnosed SSVD. Therefore, in patients with subjective cognitive impairment (SCI) or objective mild cognitive impairment (MCI) at a single memory clinic, we investigated whether serum lipid levels were associated with the risk of SSVD as well as the risk of AD or mixed dementia.

## 2 Materials and methods

### 2.1 Participants and setting

In this longitudinal study, we evaluated whether serum lipid pattern was associated with the risk of conversion from SCI/MCI to SSVD, AD or mixed dementia. The patients were recruited from the Gothenburg MCI study, a mono-center study performed at the memory clinic at Sahlgrenska University Hospital [6]. The patients underwent baseline examinations and were then followed every second year. Inclusion criteria comprised 50–79 years of age, mini mental state examination (MMSE) score > 18, and cognitive decline ≥ 6 months [6]. Exclusion criteria included acute or severe somatic diseases such as subdural hemorrhage, brain tumor, hypothyroid state, and unstable heart disease as well as psychiatric disorders such as major affective disorder, substance abuse, and confusion. In the present study, additional inclusion criteria were a baseline diagnosis of SCI or MCI, at least one follow-up visit, and a baseline determination of serum lipid levels. We excluded patients receiving lipid lowering therapy as well as patients converting to dementias other than SSVD, AD, and mixed dementia. In the final study population, all patients (*n* = 329) had values for TC and TG, whereas 326 (99 %) of the patients had values for HDL and LDL.

Totally, 817 patients were included in the Gothenburg MCI study from 1999 to 2015. Of these, 528 patients had a baseline diagnosis of SCI (*n* = 210) or MCI (*n* = 318). Then, we excluded 22 patients due to lack of baseline serum lipid determination, 89 with no follow-up visit, 23 who converted to other dementias, and 65 due to lipid lowering therapy (all these 65 patients received statin treatment). The reason for the exclusion of patients receiving lipid lowering therapy was that serum lipid levels in this group of patients may be dependent on the brand or dose of the prescribed statin. Thus, the final population consisted of 329 patients (SCI, *n* = 138 and MCI, *n* = 191).

The patients were classified using the global deterioration scale (GDS), in which GDS 1 equals no subjective or objective cognitive decline, GDS 2 corresponds to SCI, GDS 3 equals MCI, and GDS 4 is consistent with possible mild dementia [31]. The GDS score was based on medical history, checklists and evaluations of cognitive symptoms [6]: 1) Stepwise Comparative Status Analysis (STEP) [32], variables 13–20; 2) I-FLEX, a short version of the Executive Interview (EXIT) [33]; 3) MMSE [34]; and 4) Clinical Dementia Rating (CDR) [35]. Guidelines for GDS 2–3 was: STEP ≤ 1; I-FLEX ≤ 3; CDR ≤ 0.5; and MMSE ≥ 26.

The follow-up time [mean 4.1 (SD 1.8) years] was calculated from the inclusion to the date of conversion to dementia (generally at a follow-up visit) or, for those who remained stable, to the last follow-up. The maximum time of follow-up was 6 years. In patients classified as GDS 4, the specific dementia diagnoses were set by a specialized physician. The physician had access to clinical symptomatology and WMH amount (Fazekas scale [36]) on MRI, but had no knowledge of neuropsychological test results and cerebrospinal fluid (CSF) biomarker data. SSVD was defined according to the Erkinjuntti criteria [37]. Furthermore, our classification is in agreement with the Vascular Impairment of Cognition Classification Consensus Study (VICCCS), in which SSVD, denominated subcortical ischemic vascular dementia, was one of the entities [3]. More specifically: for SSVD, the patient had to have MRI-detected cerebral WMHs (mild, moderate, or severe according to Fazekas classification [36]) and predominant frontal lobe symptoms. If WMHs were only mild, then SSVD was set only if parietotemporal lobe symptoms (such as apraxia, aphasia, and agnosia) were not marked. AD was diagnosed according to the NINCDS-ADRDA criteria [38]. Mixed dementia was diagnosed if AD patients also fulfilled the criteria of SSVD [6,22]. We excluded the 23 patients who converted to other forms of dementia (cortical VaD, primary progressive aphasia, Lewy body dementia, frontotemporal dementia, or unspecified dementia).

In our total study population, 80 (24 %) of the 329 included patients converted to dementia [SSVD, *n* = 15 (5 %); AD, *n* = 39 (12 %); and mixed dementia, *n* = 26 (8 %)]. Among the patients with SCI at baseline (*n* = 138), 11 patients (8 %) converted to dementia [SSVD, *n* = 4 (3 %); AD, *n* = 3 (2 %); and mixed dementia, *n* = 4 (3 %)]. Additionally, 24 (17 %) of the 138 patients with SCI at baseline developed MCI during the follow-up. Finally, in the patients with MCI at baseline (*n* = 191), 69 (36 %) converted to dementia [SSVD, *n* = 11 (6 %); AD, *n* = 36 (19 %); and mixed dementia, *n* = 22 (12 %)].

The study was approved by the regional ethical committee in Gothenburg (diary number: L091-99 and T479-11) and the Swedish Ethical Review Authority (diary number: 2020-06733). The research was conducted according to the Declaration of Helsinki. All participants provided oral and written informed consent.

### 2.2 Assessment of covariates

Body mass index (BMI) was calculated as the weight in kilograms divided by the height in meters squared. A specialized physician recorded education level in years as well as presence of hypertension, diabetes, current medication, and smoking habits as described previously [6,22]. Hypertension was identified as antihypertensive treatment or at least two measurements of blood pressure ≥ 140/90 or ≥ 130/80 mmHg for diabetics. Diabetes was classified as fasting plasma glucose ≥ 7.0 mmol/l (126 mg/dl), two-hour plasma glucose ≥ 11.1 mmol/l (200 mg/dl) during oral glucose tolerance test, or treatment with oral antidiabetics or insulin.

### 2.3 Blood samples and cerebrospinal fluid

Blood samples were drawn in the fasted state from an antecubital vein between 08.00 and 10.00 after an overnight fast. The patients were instructed to avoid rigorous physical activity before the blood sampling, but the adherence to this instruction was not assessed. CSF samples were drawn at the lumbar vertebrae L3/L4 or L4/L5 interspace as described previously [6]. The CSF and blood samples were stored at −80 °C until assay [6].

### 2.4 Biochemical methods

All analyses were executed with the analyst being blinded to clinical information. Furthermore, all serum lipid measurements were performed using standardized methods (accredited according to international standard ISO/IEC 15189) at the Clinical Chemistry Laboratory, Sahlgrenska University Hospital, Sweden. Serum LDL level was calculated using the Friedewald formula [39]. CSF levels of total (T)-tau, phosphorylated (P)-tau 181, and β-amyloid amino acids 1 to 42 (Aβ_1–42_) were determined at the Clinical Neurochemistry Laboratory, Sahlgrenska University Hospital using sandwich ELISAs (INNOTEST, Fujirebio, Gent, Belgium). *APOE* genotype was determined by minisequencing [6].

### 2.5 Statistical analyses

The statistical analyses were performed using SPSS version 28.0 (IBM Corp., Armonk, NY, USA). Before the statistical analyses, skewedly distributed variables (all lipid variables, BMI, and CSF AD biomarkers) were logarithmically transformed. For continuous variables, a one–way analysis of variance (ANOVA) was used to assess differences across groups, followed by the Bonferroni post hoc test. In terms of the ANOVA analyses, *p*-values as well as *F*-values and partial eta square (*η*^2^) values are presented. For categorical variables, differences between groups were evaluated using chi-square tests and *χ*^2^ values, *p*-values, and partial *η*^2^ values are given. The partial *η*^2^ values are given as an index of the effect size in the comparative tests and varies between 0 and 1. Partial *η*^2^ > 0.06 indicates a medium effect size and partial *η*^2^ > 0.14 suggests a large effect size.

We used Cox proportional hazards regression to calculate hazard ratios (HRs) and 95 % confidence intervals (CIs) for the associations between serum lipid variables (TC, LDL, HDL, TG, LDL/HDL ratio, and TG/HDL ratio) and the risk of conversion to SSVD, AD, or mixed dementia. In the initial analyses, we included the lipid variables as standardized continuous variables (per SD increase). We adjusted all estimates for age and gender (model A). Furthermore, to examine the independent effect of the lipid levels on the risk of dementia, additional adjustments were made for education (years), BMI (log-transformed), current smoking (yes/no), hypertension, diabetes mellitus, and *APOE* ε4 genotype (model B). Finally, we analyzed whether the risk of SSVD was dependent on tertile of serum lipid levels using Cox proportional hazards regression. In these analyses, we compared the risk of SSVD in the lowest HDL tertile (tertile 1) vs. that in the two higher tertiles (tertiles 2–3), whereas for other lipid variables, we compared the risk of SSVD in the highest tertile (tertile 3) vs. that in the two lower tertiles (tertiles 1–2). A two-sided *p* < 0.05 was considered statistically significant.

## 3 Results

### 3.1 Clinical characteristics

Baseline characteristics of the study population (*n* = 329) are given in Table 1. There was a non-significant tendency that the proportion of men was higher in patients that later converted to SSVD. Patients that later converted to mixed dementia had higher age (*p* < 0.001) than stable SCI/MCI patients. Education (years) and BMI differed significantly across the study groups, but there were no differences between the individual groups. MMSE score was lower in patients that converted to AD (*p* < 0.001) or mixed dementia (*p* < 0.01) compared with patients with stable SCI/MCI. CSF Aβ_1–42_ level was lower in patients that converted to AD and mixed dementia compared with stable SCI/MCI patients, and CSF levels of T-tau and P-tau were higher in patients converting to AD and mixed dementia compared with patients converting to SSVD and stable SCI/MCI patients. Current smoking was higher in the patients that later converted to SSVD compared with the other study groups. The frequency of hypertension and diabetes did not differ across groups. Finally, the prevalence of *APOE* ε4 carriership was higher in patients converting to AD compared with stable SCI/MCI patients (*p* < 0.001).

**Table 1:** Baseline characteristics in patients who later converted to SSVD, AD, and mixed dementia as well as in stable SCI/MCI.

Variable	SSVD (*n* = 15)	AD (*n* = 39)	Mixed dementia (*n* = 26)	Stable SCI/MCI (*n* = 249)	*F-value or χ*^2^	*P-*value between groups	*Partial η*^2^
Men/women (n,%)	10/5 (67/33)	14/25 (36/64)	13/13 (50/50)	104/145 (42/58)	4.91	0.18	0.015
Age (years)	67.9 (6.8)	65.5 (6.8)	70.0 (6.4)^a^	63.5 (7.2)	8.41	<0.001	0.072
Education (years)	11.5 (3.5)	11.8 (3.4)	11.2 (3.6)	13.1 (3.5)	4.04	<0.01	0.037
BMI (kg/m^2^)	25.4 (3.2)	23.6 (2.7)	23.7 (3.5)	25.1 (3.5)	3.08	<0.05	0.029
MMSE score	28.3 (2.1)	27.8 (1.3)^a^	27.7 (1.6)^b^	28.7 (1.2)	8.86	<0.001	0.076
Aβ_1–42_ (ng/L)	560 (175)	460 (141)^a^	463 (176)^b^	676 (217)	7.67	<0.001	0.068
T-tau (ng/L)	327 (103)^c^^,^^d^	703 (475)^a^	640 (313)^a^	313 (149)	34.89	<0.001	0.249
P-tau (ng/L)	47.3 (8.3)^e^^,^^f^	84.9 (42.1)^a^	82.9 (40.9)^a^	53.3 (26.5)	17.06	<0.001	0.149
Smoking, yes/no (n,%)	4/11 (27/73)^e^^,^^f^^,^^g^	1/38 (3/97)	1/25 (4/96)	20/228 (8/92)	9.35	<0.05	0.029
Hypertension, yes/no (n,%)	7/8 (47/53)	8/31 (21/79)	10/16 (38/62)	66/183 (27/73)	5.39	0.15	0.016
Diabetes, yes/no (n,%)	2/13 (13/87)	2/37 (5/95)	0/26 (0/100)	8/241 (3/97)	5.37	0.15	0.016
*APOE4* allele, none/heterozygous/homozygous (n,%)	8/6/1 (53/40/7)	11/18/9 (29/47/24) a	8/12/3 (35/52/13)	140/77/15 (60/33/7)	22.34	0.001	0.069

Values are given as means (SD). All patients had subjective or objective mild cognitive impairment (SCI/MCI) at baseline. APOE ε4 genotyping was not performed in 21 patients. Differences across groups were assessed using analysis of variance (ANOVA) followed by the Bonferroni post hoc test for continuous variables and using chi-square tests for nominal variables. *F*-values were calculated using ANOVA and *χ*^2^ values were obtained using chi-square tests. Partial eta square (*η*^2^) values were assessed using ANOVA or using chi-square tests.

a *p* < 0.001 vs. stable SCI/MCI.

b *p* < 0.01 vs. stable SCI/MCI.

c *p* < 0.001 vs. AD.

d *p* < 0.01 vs. mixed dementia.

e *p* < 0.01 vs. AD.

f *p* < 0.05 vs. mixed dementia.

g *p* < 0.05 vs. stable SCI/MCI.

### 3.2 Serum lipids at baseline

At baseline, patients that later converted to SSVD had lower serum HDL and higher TG/HDL ratio compared with the other study groups (Table 2). There was also a non-significant tendency that serum TG was higher in patients that later converted to SSVD compared with the other groups. Serum levels of TC, LDL, and LDL/HDL ratio did not differ across groups (Table 2).

**Table 2:** Serum lipid pattern at baseline in patients who later converted to SSVD, AD, and mixed dementia as well as in stable SCI/MCI.

Variable	SSVD (*n* = 15)	AD (*n* = 39)	Mixed dementia (*n* = 26)	Stable SCI/MCI (*n* = 249)	*F-value*	*P*-value between groups	*Partial η*^2^
Total cholesterol (mmol/L)	5.59 (1.05)	6.03 (1.19)	5.98 (0.66)	5.74 (0.95)	1.49	0.22	0.014
LDL (mmol/L)	3.52 (0.92)	3.70 (0.95)	3.57 (0.55)	3.51 (0.89)	0.58	0.63	0.005
HDL (mmol/L)	1.33 (0.31)^a^^,^^b^^,^^c^	1.85 (0.54)	1.92 (0.60)	1.69 (0.47)	5.77	<0.001	0.051
Triglycerides (mmol/L)	1.63 (1.03)	1.05 (0.38)	1.08 (0.43)	1.19 (0.63)	2.37	0.07	0.021
LDL / HDL ratio	2.83 (1.02)	2.15 (0.81)	2.07 (0.81)	2.27 (0.95)	1.85	0.14	0.017
Triglycerides / HDL ratio	1.35 (1.09)^a^^,^^b^^,^^c^	0.63 (0.34)	0.68 (0.53)	0.82 (0.62)	4.68	<0.01	0.042

Values are given as means (SD). All patients had subjective or objective mild cognitive impairment (SCI/MCI) at baseline. All patients (*n* = 329) had values for TC and TG, whereas 326 (99 %) of the patients had values for HDL and LDL. Differences across groups were assessed using analysis of variance (ANOVA) followed by post hoc analyses using the Bonferroni post hoc test. *F*-values and partial eta square (*η*^2^) values were calculated using ANOVA.

a *p* < 0.05 vs. stable SCI/MCI.

b *p* < 0.01 vs. AD.

c *p* < 0.01 vs. mixed dementia.

### 3.3 Associations between lipids (per SD increase) and SSVD risk

During the follow-up of mean 4.1 (SD 1.8) years, 15 (4.6 %) of the patients converted to SSVD. Cox proportional hazards regression analyses were used to assess whether serum lipid levels as standardized continuous variables were associated with the risk of conversion to SSVD (Table 3). Serum levels of TC or LDL were not associated with the risk of SSVD. However, serum HDL (per SD increase) was associated with decreased risk of SSVD [base model (model A): HR 0.37, 95 % CI: 0.18–0.77; fully adjusted (model B): HR 0.35, 95 % CI: 0.15–0.79]. Furthermore, per SD increase, serum levels of TG (model A: HR 1.51, 95 % CI: 1.10–2.08; model B: HR 1.47, 95 % CI: 1.003–2.16), LDL/HDL ratio (model A: HR 1.55, 95 % CI: 1.002–2.40; model B: HR 1.68, 95 % CI: 1.03–2.74), and TG/HDL ratio (model A: HR 1.55, 95 % CI: 1.15–2.09; model B: HR 1.61, 95 % CI: 1.11–2.34) were associated with increased risk of SSVD (Table 3).

**Table 3:** The risk of conversion to dementia (SSVD, AD, or mixed dementia) per SD increase in serum lipid concentrations.

Variable	SSVD (*n* = 15)		AD (*n* = 39)		Mixed dementia (*n* = 26)	
	HR (95 % CI)	*P*-value	HR (95 % CI)	*P*-value	HR (95 % CI)	*P-*value
Total cholesterol						
Model A	0.88 (0.51–1.52)	0.65	1.28 (0.94–1.74)	0.12	1.23 (0.85–1.79)	0.28
Model B	0.99 (0.54–1.79)	0.97	1.34 (0.96–1.88)	0.09	1.07 (0.69–1.66)	0.76
LDL						
Model A	0.99 (0.58–1.69)	0.98	1.23 (0.90–1.67)	0.19	1.03 (0.70–1.51)	0.90
Model B	1.14 (0.65–1.99)	0.65	1.25 (0.89–1.77)	0.20	0.97 (0.60–1.55)	0.88
HDL						
Model A	**0.37 (0.18–0.77)**	**<0.01**	1.24 (0.91–1.70)	0.17	**1.59 (1.11–2.29)**	**0.01**
Model B	**0.35 (0.15–0.79)**	**0.01**	1.38 (0.96–1.97)	0.08	1.32 (0.82–2.14)	0.25
Triglycerides						
Model A	**1.51 (1.10–2.08)**	**0.01**	0.80 (0.53–1.19)	0.27	0.82 (0.51–1.33)	0.42
Model B	**1.47 (1.003–2.16)**	**0.048**	0.79 (0.49–1.27)	0.34	0.91 (0.53–1.56)	0.72
LDL/HDL ratio						
Model A	**1.55 (1.002–2.40)**	**0.049**	0.94 (0.66–1.32)	0.70	0.75 (0.47–1.18)	0.21
Model B	**1.68 (1.03–2.74)**	**0.04**	0.87 (0.57–1.33)	0.52	0.78 (0.45–1.36)	0.39
Triglycerides/HDL ratio						
Model A	**1.55 (1.15–2.09)**	**<0.01**	0.69 (0.43–1.12)	0.13	0.74 (0.43–1.27)	0.28
Model B	**1.61 (1.11–2.34)**	**0.01**	0.57 (0.29–1.11)	0.10	0.87 (0.49–1.53)	0.62

Hazard ratios (HRs), 95 % confidence intervals (CIs), and *p-*values were calculated using Cox proportional hazards regression analyses.

Significant associations are marked with bold text. All patients had subjective or objective mild cognitive impairment at baseline.

Model A: adjustment for age and gender.

Model B: adjustment for age, gender, education (years), BMI, current smoking (yes/no), hypertension (yes/no), diabetes mellitus (yes/no), and *APOE* ε4 allele.

### 3.4 No associations between lipids and the risk of AD or mixed dementia

Next, using Cox proportional hazards regression, we investigated whether serum lipid levels as standardized continuous variables were associated with the risk of conversion to AD (*n* = 39, 11.9%) or mixed dementia (*n* = 26, 7.9%) (Table 3). In these analyses, none of the lipid variables were associated with the risk of subsequent AD (Table 3). We found a significant association between serum HDL and the risk of mixed dementia after correction for age and gender (model A: HR 1.59, 95 % CI: 1.11–2.29), but this association lost significance after full adjustment for covariates (model B: HR 1.32, 95 % CI: 0.82–2.14). None of the other lipid variables were associated with the risk of conversion to mixed dementia.

### 3.5 Analyses of tertiles of lipids vs. the risk of SSVD

**Fig. 1:**
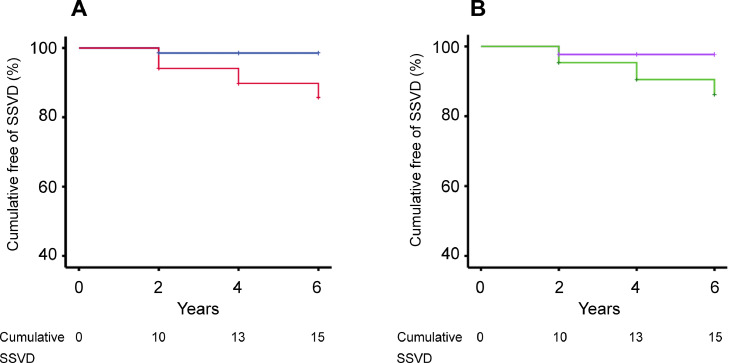
Low serum high-density lipoprotein cholesterol (HDL) and high serum triglycerides (TG)/HDL ratio are associated with increased risk of conversion to SSVD in patients with subjective or objective mild cognitive impairment. In terms of HDL, the lowest tertile was compared with the two higher tertiles, whereas for TG/HDL ratio, the highest tertile was compared with the two lower tertiles. Kaplan-Meier survival curves are presented for the risk of SSVD by (A) tertile of serum HDL concentration (log-rank test: *p* < 0.001 tertile 1 vs. tertiles 2–3) and (B) tertile of serum TG/HDL ratio (log-rank test: *p* < 0.01 tertile 3 vs. tertiles 1–2). Red, low HDL (tertile 1); blue, higher HDL (tertiles 2–3); green, high TG/HDL ratio (tertile 3); purple, lower TG/HDL ratio (tertiles 1–2).

Baseline characteristics of the study population according to tertile of serum HDL concentrations are presented in Table 4. We performed further Cox proportional hazards regression analyses to determine whether the risk of SSVD differed between tertiles of the lipid variables (Table 5). In terms of HDL, the patients in the lowest tertile had a more than sevenfold increased risk of conversion to SSVD compared with the patients in the two higher tertiles also after adjustment for covariates (tertile 1 vs. tertiles 2–3: Model A: HR 7.29, 95 % CI: 1.90–28.03; Model B: HR 7.58, 95 % CI: 1.78–32.28). Cumulative survival curves further illustrated that the risk of SSVD was dependent on tertile of serum HDL concentration (log-rank test: *p* < 0.001; Fig. 1A).

**Table 4:** Baseline characteristics in the study population according to tertile of serum HDL concentrations.

Variable	Tertile 1 (≤ 33rd percentile) (*n* = 119)	Tertile 2–3 (> 33rd percentile) (*n* = 207)	*F-value or χ*^2^	*P-*value between groups	*Partial η*^2^
Men/women (n,%)	79/40 (66/34)	60/147 (29/71)	43.22	<0.001	0.133
Age (years)	63.7 (7.1)	64.8 (7.5)	1.63	0.20	0.005
Education (years)	12.4 (3.6)	12.9 (3.5)	1.32	0.25	0.004
BMI (kg/m^2^)	26.6 (3.6)	23.8 (2.9)	54.01	<0.001	0.149
MMSE score	28.5 (1.5)	28.5 (1.3)	0.01	0.92	0.000
Aβ_1–42_ (ng/L)	620 (200)	630 (234)	0.59	0.44	0.002
T-tau (ng/L)	323 (184)	411 (297)	7.67	<0.01	0.024
P-tau (ng/L)	51.3 (22.0)	63.4 (35.8)	8.61	<0.01	0.029
Smoking, yes/no (n,%)	13/105 (11/89)	12/195 (6/94)	2.88	0.09	0.009
Hypertension, yes/no (n,%)	38/81 (32/68)	53/154 (26/74)	1.50	0.22	0.005
Diabetes, yes/no (n,%)	7/112 (6/94)	5/202 (2/98)	2.56	0.11	0.008
*APOE4* allele, none/heterozygous/homozygous (n,%)	59/45/7 (53/41/6)	107/68/20 (55/35/10)	1.90	0.39	0.000

Values are given as means (SD). All patients had subjective or objective mild cognitive impairment (SCI/MCI) at baseline. APOE ε4 genotyping was not performed in 20 patients. Serum HDL was determined in 326 patients (99 % of the total study population). Differences across groups were assessed using analysis of variance (ANOVA) followed by the Bonferroni post hoc test for continuous variables and using chi-square tests for nominal variables. *F*-values were calculated using ANOVA and *χ*^2^ values were obtained using chi-square tests. Partial eta square (*η*^2^) values were assessed using ANOVA or using chi-square tests.

**Table 5:** The risk of conversion to SSVD according to tertile of serum lipid concentrations.

	Tertile 1 (≤ 33rd percentile)		Tertile 2–3 (> 33rd percentile)
	n (%) or HR (95 % CI)	*P-*value	n (%) or HR
HDL			
SSVD, n (%)	12 (10.9 %)		3 (1.4 %)
Model A	**7.29 (1.90 – 28.03)**	**<0.01**	1.0 Referent
Model B	**7.58 (1.78 – 32.28)**	**<0.01**	1.0 Referent

	Tertile 3 (≥ 67th percentile)		Tertile 1–2 (<67th percentile)
	n (%) or HR (95 % CI)	*P-*value	n (%) or HR
Total cholesterol			
SSVD, n (%)	3 (2.7 %)		12 (5.5 %)
Model A	0.58 (0.16 – 2.06)	0.40	1.0 Referent
Model B	0.56 (0.15 – 2.13)	0.40	1.0 Referent
LDL			
SSVD, n (%)	5 (4.5 %)		10 (4.6 %)
Model A	1.05 (0.36 – 3.06)	0.94	1.0 Referent
Model B	1.17 (0.38 – 3.62)	0.79	1.0 Referent
Triglycerides			
SSVD, n (%)	8 (7.3 %)		7 (3.2 %)
Model A	**2.90 (1.04 – 8.08)**	**0.04**	1.0 Referent
Model B	2.41 (0.78 – 7.46)	0.13	1.0 Referent
LDL/HDL ratio			
SSVD, n (%)	8 (7.3 %)		7 (3.2 %)
Model A	1.90 (0.67 – 5.43)	0.23	1.0 Referent
Model B	1.99 (0.66 – 5.99)	0.22	1.0 Referent
Triglycerides/HDL ratio			
SSVD, n (%)	10 (9.1 %)		5 (2.3 %)
Model A	**3.78 (1.25 – 11.40)**	**0.02**	1.0 Referent
Model B	**3.47 (1.01 – 11.97)**	**0.049**	1.0 Referent

Hazard ratios (HRs), 95 % confidence intervals (CIs), and *p-*values were calculated using Cox proportional hazards regression analyses. In terms of HDL, the lowest tertile was compared with the two higher tertiles. For other lipid variables, the highest tertile was compared with the two lower tertiles. Significant associations are marked with bold text. All patients had subjective or objective mild cognitive impairment at baseline. Moreover, all patients (*n* = 329) had values for TC and TG, whereas 326 (99 %) of the patients had values for HDL and LDL.

Model A: adjustment for age and gender.

Model B: adjustment for age, gender, education (years), BMI, current smoking (yes/no), hypertension (yes/no), diabetes mellitus (yes/no), and *APOE* ε4 allele.

For other lipid variables, the highest tertile was compared with the two lower tertiles (Table 5). The Cox proportional hazards regression analyses showed that patients with serum TG/HDL ratio in the highest tertile had a more than threefold increased risk of SSVD compared with those in the two lower tertiles (tertile 3 vs. tertiles 1–2: model A: HR 3.78, 95 % CI: 1.25–11.40; model B: HR 3.47, 95 % CI: 1.01–11.97). Patients in the highest serum TG tertile had a significantly increased risk of SSVD after adjustment for age and gender (HR 2.90, 95 % CI: 1.04–8.08), but this association lost statistical significance after full correction for covariates (HR 2.41, 95 % CI: 0.78–7.46). For other lipid variables, the risk of SSVD in the highest tertile was similar as that in the two lower tertiles (Table 5). Finally, cumulative survival curves confirmed that the risk of SSVD was dependent on tertile of serum TG/HDL ratio (log-rank test: *p* < 0.01; Fig. 1B).

## 4 Discussion

This is the first study that has investigated the association between serum lipid concentrations and the risk of conversion to SSVD at a single memory clinic. We also evaluated whether serum lipid levels were associated with the risk of AD or mixed dementia (combined AD and SSVD). Our results show an association between higher serum HDL levels (per SD increase) and reduced risk of SSVD. Furthermore, high serum levels of TG, LDL/HDL ratio, and TG/HDL ratio were associated with increased risk of conversion to SSVD. In the following analyses of tertiles, low serum HDL and high TG/HDL ratio were associated with increased risk of subsequent SSVD. All these associations remained significant after full adjustment for multiple covariates including education (years), which was included as a covariate in the analyses since high education level may offer protection from cognitive dysfunction and development of dementia [5,6]. In contrast, after full adjustment for covariates, there were no associations between lipid levels and the risk of conversion to AD or mixed dementia.

In the present study, a major finding is that low serum HDL was associated with increased risk of conversion to SSVD. Even if the confidence intervals were relatively wide, the patients in the lowest tertile of serum HDL had a more than seven times higher risk of converting to SSVD compared with the patients in the two higher HDL tertiles. In a previous 2-sample mendelian randomization study, genetic predisposition to higher HDL was associated with lower risk of small vessel stroke and reduced WMH volume [40]. Furthermore, there are some epidemiological data suggesting that midlife HDL levels are associated with the volume and progression of WMHs [41,42]. In contrast, in a review and meta-analysis, there were no associations between late life measurements of HDL and the risk of VaD as classified using NINDS-AIREN and other criteria [10,15]. However, it has previously not been determined whether serum HDL is associated with the SSVD subtype of VaD in a memory clinic population. Therefore, our results extend the previous knowledge by showing that lower serum HDL levels are associated with increased risk of clinically manifest SSVD.

Our results showed an independent association between high serum TG levels (per SD increase) and increased risk of conversion to SSVD. In the analyses of tertiles, the association between high serum TG and increased SSVD risk lost statistical significance after full adjustment for covariates, whereas serum TG/HDL ratio in the highest tertile was still significantly associated with a more than threefold increased risk of SSVD. These findings are in line with the combined results of two population-based studies (*n* = 1842 and *n* = 766), which demonstrated that higher TG concentration was associated with higher WMH amount [28]. Furthermore, in the French Three-City cohort (*n* = 7087), higher TG levels were associated with increased risk of all-cause dementia and VaD [14]. Although the role of late life TG levels in the development of cognitive decline is not clear [43], our results suggest that high TG levels are moderately associated with increased risk of conversion to SSVD in a memory clinic population.

The constellation of low serum HDL and high serum TG is a typical feature of the metabolic syndrome [44], which in turn has been linked to increased risk of large vessel atherosclerosis [44], VaD [14,45], and increased amount of WMHs [24,46]. However, the importance for SSVD development has not been elucidated in detail. In SSVD, there are vascular pathologies such as arteriolosclerosis, edema, and damage to the blood-brain barrier (BBB) [7]. These aberrations cause chronic leakage of fluid and macromolecules into the white matter with subsequent demyelination [7]. It seems reasonable that low HDL levels can accelerate the development of SSVD as HDL participates in reverse cholesterol transport and in addition, HDL receptors in the brain microvasculature induce protective actions such as vasodilatory, anti-inflammatory, and anti-oxidative effects [40,47]. Furthermore, high TG levels could result in faster SSVD progression as high TG levels have been associated with inflammatory markers, endothelial cell inflammation, BBB dysfunction, and decreased compliance of small arteries [28,43].

We did not find any association between serum TC and LDL levels and the risk of conversion to SSVD. Although higher TC and LDL levels have been associated with increased risk of large vessel atherosclerosis [44], several population-based studies have in contrast shown inverse relationships between TC and LDL levels and the risk of subcortical small vessel disease. Although high LDL was unrelated to periventricular hyperintensities in one study (*n* = 253) [25], the combined results of two population-based studies (*n* = 1842 and *n* = 766) exhibited a negative correlation between LDL and WMH volume [28]. Furthermore, LDL level was inversely related to WMH grade in the Cardiovascular Health Study (*n* = 303) [29], and prestroke hyperlipidemia was associated with reduced WMH severity at the time of stroke [30]. Finally, in a cross-sectional memory clinic study, TC and LDL levels were reduced in manifest SSVD compared with cognitively healthy controls [22]. Thus, there are some previous indications that TC and LDL levels are negatively associated with WMH volume (a proxy of SSVD), and as cholesterol participates in the maintenance of the central nervous system [26], there is a possibility that low cholesterol could increase the vulnerability of the brain white matter [26]. However, in the present study, the lack of association between serum TC and LDL levels and the subsequent risk of SSVD may speculatively suggest that low TC and LDL levels are not a primary event in SSVD development. Instead, it could be hypothesized that the low TC and LDL levels previously found in subcortical small vessel disease are a consequence of increased cholesterol consumption due to the increased load of white matter damage.

In the present study, in which patients receiving lipid lowering therapy were excluded, there was no association between any lipid level and the risk of conversion to AD. These results are in accordance with the results of epidemiological studies showing that the associations between midlife lipid disturbances and increased risk of AD [9,10,17] are less consistent and relatively weak when the lipid measurements have been performed late in life [10,14,17]. There are inconclusive data in terms of the usefulness of lipid lowering therapy, but the results of some studies have suggested that statin treatment may reduce the risk and progression of AD [48,49]. Overall, our results combined with the earlier results could suggest that intervention strategies, such as physical exercise or medical treatment, will have larger impact on AD development and progression when employed earlier in life. Furthermore, previous studies have shown inconsistent and sometimes contradictory results in terms of the association between statin use and WMH volumes [50,51], and little is known whether statin treatment affects the progression of SSVD [50,51]. However, based on the associations between low HDL and high TG/HDL ratio and increased risk of conversion to SSVD in the present study, it could be hypothesized that intervention strategies could be valuable in SSVD also when initiated relatively close to the onset of the manifest disease.

It is unclear why low serum HDL and high serum TG were associated with increased risk of SSVD, whereas after full adjustment for covariates, there were no associations between serum lipid levels and the risk of mixed dementia (combined AD and SSVD). However, these findings are in line with the results of an earlier cross-sectional study, which found serum lipid disturbances in manifest SSVD but not in manifest mixed dementia [22]. Moreover, several other studies have demonstrated that AD neuropathology is more correlated with the clinical manifestations than coexistent cerebral subcortical small vessel disease [52,53]. Overall, further studies are needed in mixed dementia to determine the importance of the coexistent vascular pathologies for the clinical phenotype.

In the present study, we included patients with SCI (*n* = 138) and MCI (*n* = 191) at baseline (totally *n* = 329). However, the definition and assessment methods for SCI as well as the association between SCI and the risk of MCI or manifest dementia vary among studies [[54], [55], [56]]. In our study, 11 (8 %) of the patients with SCI at baseline converted to dementia [SSVD, *n* = 4 CE, *n* = 3; and mixed dementia, *n* = 4]. In addition, 24 (17 %) of the SCI patients developed MCI during the follow-up. As expected, the conversion rate in the SCI patients was clearly lower than that in the 191 patients with MCI at baseline, in whom 69 (36 %) converted to dementia. However, even so, our results provide some further support for the notion that the SCI entity is associated with an elevated risk of subsequent MCI or manifest dementia.

The present study has several strengths and limitations. Strengths include the mono-center design and the extensive characterization of the patients. As we excluded all patients with stroke-related VaD (cortical VaD), we had one study group only comprising SSVD patients. Limitations include that the number of patients that converted to dementia was small, which could have reduced the statistical power. Therefore, multi-center studies are warranted to have larger populations of patients converting to SSVD. Finally, all participants were patients seeking help at a memory clinic, which may reduce the generalizability of our findings.

## 5 Conclusions

In this mono-center study of patients with SCI or MCI, we demonstrate that low serum HDL and high serum TG/HDL ratio are associated with increased risk of conversion to SSVD. There was also an association between serum TG (per SD increase) and the risk of SSVD. Serum TC and LDL levels were not associated with the risk of SSVD, and none of the lipid variables were associated with the risk of AD or mixed dementia after full adjustment for covariates. Overall, these findings support that SSVD is a specific disease entity. In addition, our results suggest that it is of value to monitor serum lipid pattern in SCI/MCI patients at high risk of SSVD. Further studies are needed to evaluate if improvement of serum HDL and TG levels by lifestyle intervention or medical treatment can prevent or delay the development of SSVD also relatively close to the onset of the manifest disease. In contrast, in AD and mixed dementia, it seems likely that such interventions have to be initiated in earlier disease stages.

## Ethical approval

The study was approved by the regional ethical committee in Gothenburg (diary number: L091-99 and T479-11) and the Swedish Ethical Review Authority (diary number: 2020-06733). The research was conducted according to the Declaration of Helsinki. All participants provided oral and written informed consent.

## CRediT authorship contribution statement

**Elin Axelsson Andrén:** Writing – review & editing, Writing – original draft, Project administration, Methodology, Investigation, Formal analysis, Data curation, Conceptualization. **Dewa Safi:** Writing – review & editing, Formal analysis. **Anders Wallin:** Writing – review & editing, Resources, Methodology, Investigation, Funding acquisition, Conceptualization. **Johan Svensson:** Writing – review & editing, Resources, Project administration, Methodology, Investigation, Funding acquisition, Formal analysis, Data curation, Conceptualization.

## Declaration of competing interest

none.
